# Bio-degumming technology of jute bast by *Pectobacterium* sp. DCE-01

**DOI:** 10.1186/s13568-016-0255-3

**Published:** 2016-10-03

**Authors:** Shengwen Duan, Xiangyuan Feng, Lifeng Cheng, Yuande Peng, Ke Zheng, Zhengchu Liu

**Affiliations:** Institute of Bast Fiber Crops, Chinese Academy of Agriculture Sciences, Changsha, 410205 China

**Keywords:** Bio-degumming, *Pectobacterium* sp., Jute, Fiber

## Abstract

Among industrial fiber crops, jute is ranked second to cotton in terms of yield and planting area worldwide. The traditional water retting and chemical semi-degumming methods restrict the development of the jute industry. Jute fiber can be extracted from jute bast through mechanical rolling (preprocessing), culture of bacteria, soaking fermentation (liquor ratio = 10, inoculum size = 1 %, temperature = 35 °C, and time = 15 h), inactivation, washing, and drying. *Pectobacterium* sp. DCE-01 secretes key degumming enzymes: pectinase, mannase, and xylanase, which match well the main non-cellulosic components of jute bast. Compared with the traditional water retting degumming, the bio-degumming cycle is shortened from more than 10 days to 15 h. The proposed bio-degumming achieved higher efficiency and lower pollution than water retting and chemical semi-degumming.

## Introduction

Jute belongs to the genus *Corchorus* in family *Tiliaceae*. This annual herbaceous industrial crop is ranked second to cotton in terms of yield and planting area worldwide. Jute is mainly produced in India, Bangladesh, China, Thailand, Brazil, and Australia. *Corchorus capsularis* L. and *Corchorus olitorius* L. are the common jute cultivars (Ahmed and Akhter 2001; Haque et al. 2003). A jute fiber is shiny white and displays good hygroscopicity and rapid water apron. The high biomass and excellent quality of jute fiber are comparable to those of woods. Fiber is an important raw material for packages, ropes, carpets, and canvasses. In recent years, fiber has widely attracted attention as a multipurpose, renewable, and environment-friendly natural resource. The United States, Japan, India, China, Germany, and Australia have developed various products in bast fiber spinning design, papermaking, building materials, jute–plastic composite, adsorption, forages, biological energy sources, culture medium, and other fields (Chen and Yang 2013; Xiong 2008).

Cellulose, hemicellulose, pectin, lignin, cerolipoid, and ash are the main components in jute bast. The cellulose content of jute bast is 59–63 %, and the other non-cellulosic components are called gum. Jute bast displays high lignin content, reaching up to 11–16 %. A jute fiber is considerably short, and the gum binds jute fibers together. Only jute fibers with the most amount of eliminated gum can be used as raw materials for spinning (Haoran 1993).

The disadvantages of traditional water retting include serious pollution, long degumming time, poor quality and stability, and high labor intensity. Chemical semi-degumming is disadvantageous because it causes heavy pollution and severe fiber damages; moreover, this method is costly. Chemical semi-degumming is inapplicable to jute fibers, which are short and existing as bundles. Therefore, the imperfect degumming technology restricts the development of the jute industry (Ahmed and Akhter 2001; Banik et al. 2007). Bio-degumming catalyzes and degrades non-cellulosic components (including pectin, hemicelluloses, and lignin) in jute bast by using enzymes secreted by microorganisms, resulting in cellulose extraction (Kozlowski et al. 2006). Bio-degumming is a clean, environmentally friendly, highly efficient, high-quality, and low-cost method and is becoming the leading jute degumming technology (Banik et al. 2007; Biswas et al. 2013; Liu and Peng 2004). Microbial degumming involves enzyme degumming and bacterial degumming. However, no research on enzyme catalytic mechanism on bio-degumming of jute has been reported. Moreover, the immature enzyme mixing technology and the costly enzymes restrict the applications of enzyme degumming. For bacterial degumming, some researchers have studied the screening and community structure of degumming bacterial strains of jute (Banik et al. 2007; Das et al. 2012; Haque et al. 2003; Munshi and Chattoo 2008). A mixed bacterial retting culture was inoculated during ribbon retting of jute, and most of the defects arising from conventional retting could be overcome by ribbon retting (Banik et al. 2003). An ecofriendly and water saving retting technology of jute has been developed by fermentation by fermentation procedure (Banik 2016).

To accelerate the further development of the jute degumming technology, after pre-processing through mechanical rolling, a set of optimized bio-degumming technology for jute was developed using the highly efficient *Pectobacterium* sp. DCE-01.

## Materials and methods

### Raw material

Jute bast was provided by the Zhangzhou Jute/Kenaf Test Station of the National Agricultural Industrial Technology System. The jute bast from *Corchorus capsularis* Zhonghuangma No. 1 at 110 days of growth was dry and free of mildew. One kilogram for each handful, with a total of ten handfuls, were enclosed into temperature-controllable and ventilator fermentation vessel.

### Strain

*Pectobacterium* sp. DCE-01 (CGMCC5522) was used in this study. This strain was cultivated and collected by the authors.

### Strain culture

The *Pectobacterium* sp. DCE-01 strain was cultivated at 34 °C for 6 h under the velocity of 180 rpm. The medium consists of 1.0 % glucose, 0.5 % NaCl, 0.5 % beef extract, 0.5 % peptone, and 100 mL of water.

### Design of bio-degumming technology and optimization of fermentation conditions

#### Preprocessing

Jute bast was exposed to sunlight for 1–2 days. The head and tail (20 cm) of the jute bast were cut. After eliminating the dusts and impurities, the jute bast was preprocessed through mechanical rolling (Liu et al. 2012).

#### Fermentation degumming

Different fermentation conditions were set, including temperature (31–37 °C), bath ratio (1:10–1:25), fermentation time (5–20 h), and inoculum size (1–4 %). The oscillation velocity of the shaker was fixed at 180 rpm. Different orthogonal test factors and levels were set (Table 1). The orthogonal test results were statistically analyzed using IBM SPSS 22.0 (SPSS Inc., Chicago, USA).

**Table 1:** Orthogonal test factors and levels of fermentation parameters

Level	A	B	C	D
	Inoculum size (%)	Temperature (°C)	Bath ratio	Time (h)
1	1.0	31	1:10	5
2	1.5	33	1:15	10
3	2.0	35	1:20	15
4	2.5	37	1:25	20

Inactivation and jute washing: The jute was boiled in water at 105 °C for 20 min immediately after degumming and then washed using a bast fiber washing machine.

### Comparative analysis of different degumming technologies

Traditional water retting, chemical semi-degumming, and optimized bio-degumming technologies were compared. Their technical routes were described as follows:

1. Bio-degumming: jute bast → preprocessing → soaking fermentation → inactivation → washing with water → dehydration → drying → jute fiber.
2. Water retting: jute bast → natural soaking → washing with water → drying under the sun → jute fiber.
3. Chemical semi-degumming: jute bast → preprocessing → pickling (1 mL/L, 55 °C, 1:15, 60 min) → washing with water → digestion (16 g/L (mass fraction) NaOH, 3 g/L Na_2_SiO_3_, 4 g/L Na_2_SO_3_, 2 g/L penetrant, 90 °C, 1:20, 120 min) → washing with water → pickling → washing with water → dehydration → drying → jute fiber (Wang and Cai 2008).

### Test for effects of degumming

Removal rate of non-cellulose: Jute bast (M_0_) was dried into constant weight before degumming, followed by fermentation and washing. The jute bast was subsequently dried into constant weight (M_1_). The weight loss ratio was V = (M_0_ − M_1_)/M_0_ × 100 % (Zeng and Xiang 2007). Fiber fineness is detected with weighing method while fiber strength is detected with single-fiber strength tester (Fu et al. 2008).

Residual gum content was tested by referring to the quantitative analysis of the chemical components of ramie (Jiang and Shao 1986).

Chemical oxygen demand (COD) test: COD concentration in fermentation liquor after degummed by COD detector ET99718 (Lovibond^®^ Group, Germany) was tested according to specifications (Han et al. 2013).

All the test data were repeated three times, and got the average of three replicates.

### Enzyme activity during degumming

Enzyme activity is defined as the enzyme quantity needed to degrade 1 μmol of substrate per minute. The enzyme activity of strains was tested using the dinitrosalicylic acid method (Do et al. 2016; Sohail et al. 2016; Wang 2009).

### Monosaccharide test during degumming

During fermentation (0–15 h) of the bio-degumming of jute bast, samples of fermentation liquid liquor were collected every 3 h and then separated by a 0.2 μm membrane (Vivaflow 200). The filtered solution was used to test the monosaccharide content. The monosaccharide components generated after jute hydrolysis were systematically analyzed using 1-phenyl-3-methyl-5-pyrazolone precolumn derivatization in high-performance liquid chromatography (HPLC). Monosaccharides and their derivative components and contents in the hydrolysate were tested by C_18_ chromatographic analysis and ultraviolet detection, after jute acetolysis was detected using trifluoroacetic acid (Zhang et al. 2013; Fang et al. 2015).

### Change rule of living bacterium content, pH and oxidation reduction potential

During jute degumming process, the quantity of degumming bacterial strain DCE-01 was determined through plate bacterial colony counting method (Shen and Chen 2007). pH and ORP were determined with automatic potential determinator ZDJ-4A from INESA instrument (Shanghai, China).

## Results

### Fermentation parameters for jute bio-degumming

**Fig. 1:**
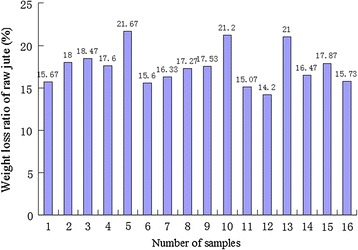
Weight loss ratio of raw jute bast under different fermentation conditions

Figure 1 shows the results of the test for weight loss ratio of raw jute bast (V) under different fermentation conditions.

The results of analysis of variance and multiple comparison are listed in Tables 2 and 3, respectively. The analysis of variance (Table 2) shows that bath ratio significantly influenced the weight loss ratio of jute, whereas inoculum size, temperature, and time slightly affected the weight loss ratio of jute. This phenomenon was observed possibly because the bath ratio influenced the oxygen content of the fermentation liquor; however, high bath ratio inhibited the growth of the degumming strain *Pectobacterium* sp. DCE-01. The results of multiple comparison (Table 3) show that the bath ratio exerted the greatest influence on the weight loss ratio of jute, successively followed by inoculum size, temperature, and time. The level that least influenced the weight loss ratio of jute was considered the optimal level. Therefore, the optimal levels for jute fermentation were set as follows: bath ratio = 10, inoculum size = 1 %, temperature = 35 °C, and time = 15 h.

**Table 2:** Analysis of variance of fermentation parameters in bio-degumming of jute

	F	P
Inoculum size	0.853	0.551
Temperature	0.875	0.542
Bath ratio	33.537	0.008
Time	1.255	0.428

**Table 3:** Multiple comparisons of fermentation parameters in bio-degumming of jute

	Inoculum size	Temperature	Bath ratio	Time
K1	15.65	16.1	12.72	15.8175
K2	16.2	16.8175	15.5	16.5825
K3	16.3175	15.785	18.085	16.735
K4	16.685	16.15	18.5475	15.7175
R	1.035	1.0325	5.8275	1.0175

K is index level; K1–K4 represent 1st–4th levels; R is a statistical value, representing the influences of this factor on weight loss ratio of raw jute

### Comparison of the effects of jute degumming

Table 4 shows the residual gum content in jute, weight loss ratio of raw jute, fiber breaking strength, and COD in fermentation liquor under different degumming technologies.

**Table 4:** Jute degumming effects of different degumming technologies

	Residual gum content (%)	Weight loss ratio of raw jute (%)	Fiber breaking strength (cN d/tex )	Fiber fineness	COD (mg/L)
Bio-degumming	20.15	18.21	5.12	3.2	3870
Water retting	22.37	22.15	3.06	3.0	5260
Chemical semi-degumming	17.52	26.43	4.48	2.4	7530

The residual gum content in water retting was higher by 22.37, 11.01 and 27.68 % than those of bio-degumming and chemical semi-degumming. The residual gum content of bio-degumming was 15.01 % higher than that of chemical semi-degumming, indicating that this chemical reagent can dissolve gum more thoroughly than biological enzymes secreted by the *Pectobacterium* sp. DCE-01; however, the amount of microorganism for jute degumming during water retting is inadequate. The weight loss ratio of bio-degumming was lower by 17.79 and 31.10 % than those of water retting and chemical semi-degumming, respectively. The residual gum content and weight loss ratio of water retting were high, whereas the fiber breaking strength decreased significantly, indicating that fiber degradation strains exist in the natural environment, thereby damaging the jute fibers and reducing the jute fiber quality. The fiber breaking strength of bio-degumming was 14.29 % higher than that of chemical semi-degumming, indicating that bio-degumming maintained fiber strength well. Fiber strength obtained by jute processing with DCE-01 was the highest, followed by fibers obtained by water retting, and chemical semi-degumming methods. Moreover, the COD of bio-degumming was 26.43 and 48.61 % lower than those of water retting and chemical semi-degumming, respectively.

### Enzyme activity in fermentation liquor (unit: U/mL)

During fermentation degumming of jute, the *Pectobacterium* sp. DCE-01 secreted xylanase, pectinase, mannose, and cellulase. The activities of these enzymes are presented in Table 5.

**Table 5:** Activity of enzymes secreted by *Pectobacterium* sp. DCE-01 during degumming (unit: U/mL)

	Fermentation time (h)					
	0	3	6	9	12	15
Xylanase	1.98	2.08	2.19	3.02	3.23	7.39
Pectinase	4.37	42.25	102.19	331.95	339.02	353.8
Mannase	3.02	4.16	15.61	28.41	37.36	54.84
Cellulase	0	1.12	1.92	2.43	2.76	3.05

The catalytic activity of pectinase, mannase, xylanase, and cellulase increased gradually during fermentation. After inoculation, the activity of pectinase increased by 75.9 times within 9 h and then stabilized. The activities of mannase and xylanase increased stably with time, increasing by 18.2 and 3.7 times, respectively, after 12 h of fermentation. The activity of pectinase reached up to 353.8 after 12 h of fermentation, which was 6.45 and 47.88 times higher than those of mannase and xylanase, respectively. Just after inoculation, cellulase couldn’t be detected, and then its content gradually increased. Its content at 15 h was 2.7 times of that at 3 h, but the enzyme activity was low and increasing rate was slow.

### Monosaccharide content of fermentation liquor

**Fig. 2:**
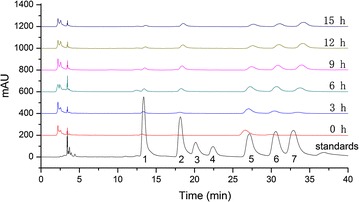
Chromatogram of monosaccharide components in the fermentation liquor of jute. *1* mannose, *2* rhamnose, *3* glucuronic acid, *4* galacturonic acid, *5* glucose, *6* galactose, *7* xylose

Mannose, rhamnose, galacturonic acid, glucose, galactose, and xylose were detected in the supernate of fermentation liquor during bio-degumming of jute; however, no glucuronic acid was detected. Figure 2 and Table 6 respectively show the liquid chromatogram and concentrations of monosaccharide components of the fermentation liquor. As fermentation proceeded, galacturonic acid increased continuously at a slow rate, whereas the other monosaccharides increased first and then decreased. After 9 h, rhamnose and xylose concentrations peaked at 37.32 and 66.75 μg/mL, respectively. In addition, the concentrations of mannase, glucose, and galactose peaked at 13.41, 78.17 and 78 μg/mL, respectively, after 6 h (Fig. 2).

**Table 6:** Concentrations of monosaccharide components in 0–10 h fermentation liquor of jute (unit: μg/mL)

	Mannose	Rhamnose	Glucuronic acid	Galacturonic acid	Glucose	Galactose	Xylose
0 h	0.84	0.19	0	0	38.3	0.69	0.58
3 h	9.7	13.59	0	0.29	72.07	38.46	17.22
6 h	13.41	25.68	0	0.35	78.17	78	53.9
9 h	10.37	37.32	0	0.51	51.51	70.69	66.75
12 h	8.37	32.64	0	0.78	42.36	57.11	65.33
15 h	7.37	28.78	0	0.9	38.24	44.51	58.58

**Table 7:** Change rule of living bacterium content, pH and ORP

	Fermentation time (h)					
	0	3	6	9	12	15
Living bacterium content	1.6 × 10^6^	3.2 × 10^6^	2.5 × 10^7^	7.9 × 10^7^	9.2 × 10^7^	7.2 × 10^7^
pH	5.91	5.99	6.12	6.37	6.85	7.23
ORP (mV)	66.3	61.2	46.8	22.7	3.6	−12.1

The initial concentration of glucose was relatively high, which may be related to glucose in the culture medium. Galacturonic acid in pectin degradation products was relatively low, although it increased continuously. This phenomenon was observed possibly because pectinase was the key enzyme, although residual pectinase was detected in the fermentation supernate resulting from the timely, thorough, and great demands of microbial degradation.

After inoculation and DCE-01 bacterial strain experienced a 3-h adaptation period, the quantity of viable bacteria rapidly increased, after 9 h of culturing, the concentration of viable bacteria increased by 49 times and then entered into a stable period. The quantity of viable bacteria started decreasing at 15 h, and the whole process presented typical bacterial growth law. Within 12 h, pH was kept acidic, but gradually increased and turned into alkalinity at 15 h. Within 12 h, ORP > 0 which meant continuous aerobic status. As culturing time passed, it weakened and then slowly rose again, and reached anaerobic status at 15 h (Table 7).

## Discussion

Technologies for bio-degumming of *Erwinia carotovora* (Liu and Peng 2004), *Bacillus pumilus* (Basu et al. 2009)*, Aspergillus niger* (Zheng et al. 2010), and *Bacillus subtilis* (Guo et al. 2011) on ramie, kenaf, and other fiber crops have been reported in China and in foreign countries. However, few studies have investigated the bio-degumming of jute. A *Micrococcus* sp. strain that can accomplish jute degumming within 6 days was screened (Haque et al. 2003). The proposed degumming technology was combined with mechanical rolling preprocessing, which can accomplish bio-degumming of jute within 15 h. This approach includes two important aspects: (1) A highly efficient degumming strain *Pectobacterium* sp. DCE-01 was reported, which secretes mannase, pectinase, and xylanase simultaneously. (2) This approach adopted roller compaction engine prior to biological fermentation. The mechanical rolling (preprocessing) disrupted the adhesion between non-cellulosic materials and cellulose, resulting in direct fall-off of abundant non-cellulosic materials, intensifying the infiltration capacity of microorganism and its enzymes. As a result, the degumming effect of jute improved significantly.

Conventional water retting of jute is characterized by high water consumption, heavy pollution, and long degumming time (Haque et al. 2002), whereas chemical semi-degumming of jute is disadvantageous in terms of heavy pollution and severe fiber damages (Ahmed and Akhter 2001). However, the bio-degumming of jute does not require strong acid and strong base and is characterized by gentle degumming technological conditions, high efficiency, high quality, and low pollution. In contrast with the ribbon retting of jute (Banik et al. 2003), during jute degumming process of bacterial strain DCE-01, within 12 h, pH is kept acidic while ORP kept being greater than 0 namely under aerobic status, at 15 h, pH presented alkaline status while ORP was negative namely under anaerobic status. During degumming process, while degrading substrate and generating acidic substances, degumming microorganisms consumed acidic substances. Inconsistency in degradation products and product utilization rate between aerobic degumming and anaerobic degumming processes resulted in two totally different degumming systems.

The gum of jute consists of complex components, including 13–20 % hemicelluloses, 11–16 % lignin, and 2 % pectin (Lu 1993; Xu et al. 2010). During degumming, the use of key enzymes that match well the jute gum is necessary. However, natural strains secreting enzymes suitable for jute gum degradation are difficult to the castle, resulting in slow development of bio-degumming. The *Pectobacterium* sp. DCE-01 simultaneously secretes pectinase, mannase, and xylanase during jute degumming, and the activities of these enzymes at 15 h reached up to 353.8, 54.84 and 7.39 U/mL, respectively. In the meantime, this bacterium also secreted cellulose, but with low enzyme activity and slow increasing rate. Just after inoculation, the cellulase couldn’t be detected, and then its content increased gradually. Its content was 1.12 U/mL at 3 h, and that at 15 h was 3.05 U/mL. Cellulase could reduce jute fiber strength, and exert negative effect on fiber performance, hence subsequent study should consider deleting this gene. During degumming, hydrolysates of jute included mannose, rhamnose, galacturonic acid, glucose, galactose, and xylose. As fermentation proceeded, galacturonic acid increased continuously at a slow rate, whereas the other monosaccharide components increased first and then subsequently decreased. This result demonstrated that the *Pectobacterium* sp. DCE-01 released pectinase, mannase, and xylanase continuously during jute degumming.
